# The Effect of Using an Inappropriate Protein Database for Proteomic Data Analysis

**DOI:** 10.1371/journal.pone.0020873

**Published:** 2011-06-14

**Authors:** Giselle M. Knudsen, Robert J. Chalkley

**Affiliations:** Department of Pharmaceutical Chemistry, University of California San Francisco, San Francisco, California, United States of America; Institute for Research in Biomedicine, Spain

## Abstract

A recent study by Bromenshenk et al., published in PLoS One (2010), used proteomic analysis to identify peptides purportedly of Iridovirus and Nosema origin; however the validity of this finding is controversial. We show here through re-analysis of a subset of this data that many of the spectra identified by Bromenshenk *et al.* as deriving from Iridovirus and Nosema proteins are actually products from Apis mellifera honey bee proteins. We find no reliable evidence that proteins from Iridovirus and Nosema are present in the samples that were re-analyzed. This article is also intended as a learning exercise for illustrating some of the potential pitfalls of analysis of mass spectrometry proteomic data and to encourage authors to observe MS/MS data reporting guidelines that would facilitate recognition of analysis problems during the review process.

## Introduction

Identification of proteins in complex biological samples through MS/MS peptide fragmentation analysis is a mature technique, supported by multiple data analysis packages [1]. The standard approach involves digesting proteins into peptides using a proteolytic enzyme, most commonly trypsin, then sequentially isolating individual peptides in the mass spectrometer, fragmenting the peptides, and measuring the masses of the resulting fragmentation products [2].

Peptides are identified by database searching strategies. Starting from a protein database containing potential proteins that could be present in the sample, these proteins are digested in silico by a search engine; e.g. if trypsin was used as the proteolytic enzyme, the search engine would calculate the masses of all peptides that could be produced by cleavage after lysine and arginine residues, to create a virtual peptide database. For identification of peptides in the sample, the search engine first filters this peptide database to determine all potential peptides that have the same mass as an observed peptide in the sample. It then performs an in silico fragmentation of each of these peptides and compares the list of fragment ions that would be expected from each of the sequences in the peptide database with the list of fragment masses observed in the fragmentation spectrum derived from a peptide in the sample. Results are scored, depending on the search engine used, on the basis of cross-correlation between theoretical and observed spectra, or using scoring systems based on empirical or statistical analysis of fragments observed in spectra. The result is a best-scoring match that may be correct or incorrect.

These scores are converted into a statistical measure such as a probability or an expectation value by either theoretical or empirical means to try to determine which assignments are reliable. For example, widely used tools for post-processing results from the search engines such as Sequest [3] are the Peptide and Protein Prophet programs [4]. These re-score results on the basis of several metrics; for example, as peptides are derived from proteins, they will give increased score to identifications of peptides present in proteins that have already been identified as being present in the sample on the basis of other peptide identifications. The software then makes the assumption that within the results there will be two distributions of scores present: scores of spectra matched to peptides that are correctly assigned and scores matched to spectra that are products of random matches. The software tries to deconvolve these two distributions to allow conversion of scores into a probability of an assignment being correct.

Having determined a score threshold to be used for reporting results a second metric, a false discovery rate (FDR), can be calculated that measures the reliability of a set of results as a whole. The standard approach to determine this global error rate is to search data against a decoy database of the same size as the one queried for peptide and protein identification, but one that does not contain any correct peptide sequences. The most common way to create such a database is to shuffle or reverse the sequences present in the normal database. Based on the number of spectral matches to peptides in this decoy database above a given threshold score it is possible to estimate the number of random matches in the results from the target normal database [5].

Unreliable results can be produced by the use of an inappropriate database, incorrect search engine parameters, or employment of an unsuitable acceptance score threshold. As a result, the proteomics community has outlined a series of publication guidelines that describe minimal information required in order to allow independent assessment of MS proteomics results [6], [7]. They also encourage the deposition of raw MS data sets in public repositories such as Tranche [8] that allows independent re-analysis of data.

In this manuscript, we show that the identifications of Iridovirus and Nosema in three representative honey bee samples reported by Bromenshenk *et al.* [9] resulted from the use of an inappropriate database.

## Results

Searching the honey bee-derived protein sample data against all species in the NCBI non-redundant database resulted in the identification of seventy to ninety previously unreported Apis mellifera honey bee proteins in each sample (Supporting Information S1). In addition to these honey bee identifications, highly conserved proteins such as actin, tubulin, ribosomal subunits and heat shock proteins were matched to other insect species such as Nasonia vitripennis, Drosphila melanogaster, and Bombyx morii. These are likely mis-identified species that should belong to honey bee proteins, but could have been missed due to incomplete sequence information for the Apis genus in the NCBInr database. Finally, a few proteins were identified from unrelated organisms including tick, tuberculosis, and spider; however, these proteins were identified based on one- or two-peptide matches and cannot be expected to be reliable species identifications. The only exception is the identification of human keratin peptides, which are common laboratory contaminants.

**Figure 1:**
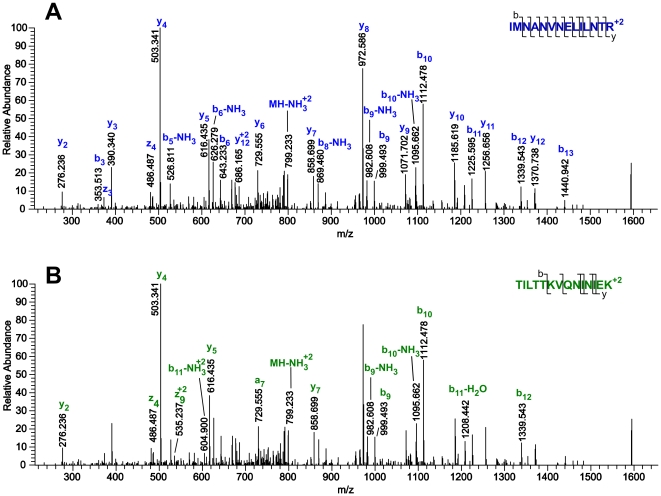
Example of a reassigned spectrum, with m/z 803.3 (2+) at 102.371 minutes in sample ECBC_Bees03. A) Assignment to peptide sequence IMNANVNELILNTR^2+^ from Acc#58585098 *Apis mellifera* major royal jelly protein 1. B) The published assignment to TILTTKVQNINIEK^2+^ from Acc# NP_149513.1 from *Iridovirus IIV6* protein 050L.

Furthermore, these searches did not match any peptide spectra to either Nosema or Iridovirus, the major species previously reported [9]. Nosema and Iridovirus are both well represented in the NCBInr database, with 2135 and 505 entries respectively, compared with 9,746 Apis mellifera entries. Over one third of the spectra matched in the previous report were automatically reassigned to highly abundant honey bee proteins in the Protein Prospector searches, reported in Supporting Information S2. An example of a reassigned spectrum is shown in Figure 1. Increasing the precursor mass error tolerance, or reducing the database to sequences only from the Apis genus allowed for a few additional peptide identifications (indicated in italics in Supporting Information S2). Seventy-four out of 172 spectra previously matched to Nosema or Iridovirus were reassigned in this analysis. The other spectra did not return a confident identification, despite considering Nosema and Iridovirus proteins. In all proteomic analyses there are a significant number of spectra acquired that cannot be reliably assigned, so having many unassigned spectra is normal.

## Discussion

Including all relevant species in database searches for these samples from the Bromenshenk *et al.* study [9] has resulted in the corrected identification of peptides derived from several highly abundant honey bee proteins such as mellitin, vitellogenin and major royal jelly proteins. These peptide identifications were predictable, because honey bee proteins would be expected to be orders of magnitude greater in abundance over any microbial pathogens that may have been present in the original honey bee samples, and are corroborated by the matching of several peptides to a single protein rather than single peptide identifications reported in the previous analysis.

At issue in the previous analysis is the usage of a highly restricted database of only 978 entries. Over half of these entries were Iridovirus, and no honey bee sequences were included. This is a case where the journal publication guidelines in the proteomics community [6],[7] would have redirected the analysis before publication of false identifications. They clearly state: “If the database or library used is very small (<1000 entries) or excludes common contaminants, justification must be specifically provided since this may generate misleading assignments and an inaccurate false discovery rate estimate.”

All the identifications reported in the previous analysis of these samples had Protein/Peptide Prophet probabilities greater than 0.95. The reason for these grossly inaccurate probability estimates is that Peptide Prophet makes the assumption that there are correct answers among those submitted to it for analysis. If this is not the case it ends up modeling the highest scoring incorrect answers as being reliable assignments. If the authors had tried to calculate a FDR for their dataset by searching against a decoy database, the problem would have been immediately apparent, as they would have observed as many matches to the decoy database as to the target normal database. Target-decoy database searching is easy to perform, so is a sensible step in all proteomic analyses to get a second independent measure of reliability to results independent of the probabilities reported by other software.

A recent article by Foster [10] addressed the high probability of a high false discovery rate in the Bromenshenk study, even in the absence of having access to raw data files. The analysis here agrees strongly with Foster's arguments that were based on the logic of protein abundance and the frequency of missed trypsin cleavages reported in the Bromenshenk study. The analysis here identified peptides with missed trypsin cleavages of ∼17% for single missed cleavages and ∼5% double missed cleavages, consistent with Foster's analysis.

We conclude that there is no evidence for the presence of Iridovirus or Nosema peptides in a representative set of data from the Bromenshenk study, and that most if not all previously identified peptides can be explained as deriving from highly abundant honey bee proteins. This does not preclude evidence from other work, such as genomic sequencing efforts (Runckel, Flenniken *et al. manuscript under review*), which do support the presence of Nosema in similar samples. The use of a severely restricted database that excluded honey bee sequences in the previous study seriously draws into question their evidence of linkage between colony collapse disorder and the presence of Iridovirus and Nosema infection.

## Methods

Three representative sample files were analyzed from the Bromenshenk *et al.* study and these have been made publicly available through deposition in Tranche at ProteomeCommons.org (data may be downloaded using the following hashes: 0BSo6r0GEZffeibHTbbdfkoQah4QIgQyfbrPR8NVqSY5/RD5GBguMg6PgYF5ZX/RtaKn0eove2FUZjhSUWR7FOYbCX0AAAAAAAAEEA =  = ). Previous analysis of these data was reported in a supplementary report titled ECBC-TR-814, obtained through the editors of PLoS One, and in this report the results for ECBC-Bees02, ECBC-Bees03 and ECBC-Bees04 were identified as Test 10, Test 34 and Test 32 respectively.

Data were processed and database searched using Protein Prospector v. 5.7.1 (http://prospector.ucsf.edu/prospector/mshome.htm). Data were searched against all species in the NCBI non-redundant database from 6/17/2010, since the samples were prepared from whole honey bee soluble lysates and were hypothesized to contain multiple microbes [9]. For false discovery rate estimation, this database containing 11,205,216 entries was concatenated with a duplicate database containing 11,205,216 randomized entries, for a total of 22,410,432 entries in the final database [5].

Peptide matching was performed using trypsin as the digestion enzyme, and mass accuracy was set at 0.8 Da for both parent and fragment masses. Searches were performed allowing for one non-specific trypsin cleavage per peptide and two missed cleavages, as no protease inhibitors were included in the sample preparation reported. Up to two variable modifications to side-chains were allowed from the following list: acetyl (protein N-term), acetyl+oxidation (protein N-term Met), Gln→pyro-Glu (N-term Gln), Met-loss (protein N-term Met), Met-loss+acetyl (protein N-term Met), oxidation (Met), oxidation (Trp), and dioxidation (Trp). Threshold values for reporting protein and peptide identifications from Protein Prospector searches were: minimum protein score 22, minimum peptide score 15, maximum E-value protein 0.01, and maximum E-value peptide 0.05. FDR at this threshold was estimated at 2.4% for proteins and 0.7% for peptides.

## Supporting Information

Supporting Information S1 **Protein prospector search results for ECBC_02 (Proteins and Peptides identified in Tables S1 and S2, respectively), ECBC_3 (Proteins and Peptides identified in Tables S3 and S4, respectively), and ECBC_4 (Proteins and Peptides identified in Tables S5 and S6, respectively).**
(XLS)

Supporting Information S2 **Re-analysis of ECBC_02, reported previously as Test 10 (Table S7), ECBC_03, reported as Test 34 (Table S8), and ECBC_04, reported as Test 32 (Table S9).**
(XLS)

## References

[1] Nesvizhskii AI (2010). A survey of computational methods and error rate estimation procedures for peptide and protein identification in shotgun proteomics.. Journal of Proteomics.

[2] Aebersold R, Mann M (2003). Mass spectrometry-based proteomics.. Nature.

[3] Eng JK, Mccormack AL, Yates JR (1994). An Approach to Correlate Tandem Mass-Spectral Data of Peptides with Amino-Acid-Sequences in a Protein Database.. Journal of the American Society for Mass Spectrometry.

[4] Keller A, Nesvizhskii AI, Kolker E, Aebersold R (2002). Empirical statistical model to estimate the accuracy of peptide identifications made by MS/MS and database search.. Analytical Chemistry.

[5] Elias JE, Gygi SP (2007). Target-decoy search strategy for increased confidence in large-scale protein identifications by mass spectrometry.. Nat Methods.

[6] Orchard S, Jones P, Taylor C, Zhu W, Julian RK (2007). Proteomic data exchange and storage: the need for common standards and public repositories.. Methods Mol Biol.

[7] Revised Publication Guidelines for Documenting the Identification and Quantification of Peptides, Proteins, and Post-Translational Modifications by Mass Spectrometry.. http://mcponline.org/site/misc/PhialdelphiaGuidelinesFINALDRAFT.pdf.

[8] Falkner JA, Hill JA, Andrews PC (2008). Proteomics FASTA archive and reference resource.. Proteomics.

[9] Bromenshenk JJ, Henderson CB, Wick CH, Stanford MF, Zulich AW (2010). Iridovirus and microsporidian linked to honey bee colony decline.. PLoS One.

[10] Foster LJ (2011). Interpretation of data underlying the link between CCD and an invertebrate iridescent virus.. Mol Cell Proteomics.

